# Book Reviews

**Published:** 1819-04

**Authors:** 


### CRITICAL ANALYSIS

### OF RECENT PUBLICATIONS,

IN THE

### DIFFERENT BRANCHES OF MEDICINE, SURGERY, &c.

*Transactions of the Association of Fellows and Licentiates of the King's and Queen's College of Physicians in Ireland.* Vol. II.—8vo. pp. 600. Cummings, Dublin; and Longman and Co. London.

WE entered on the perusal of this contribution to medical science with the most interesting and grateful sensations, from the recollection of the information we had derived from the former part of the Transactions of the same learned Society; and the expectations we had formed respecting its value have not terminated in disappointment. Transactions of Societies constituted, like the above, of men really eminent for talents, furnish a rich intellectual banquet to the studious enquirer, who is disposed to recognize the superiority of important facts to the most plausible hypotheses merely founded on probable data: a disposition which we all assume, after a certain extent of experience and reflection have tempered the ardour of youthful expectation. This principle is promulgated in the institutes of all the most eminent scientific societies in Europe, which require in the contributions of their members, a detail of facts judiciously selected and accurately arranged, devoid of hypotheses, and, indeed, of any theoretical disquisitions beyond what may be necessary to elucidate and develop the circumstances to which they immediately relate: requisitions that may be but little flattering to indolence, vanity, and a vivid imagination, but which are eminently favourable to the discovery of truth, and the certain, though perhaps slow, advancement of useful knowledge. The dictates of such institutes, relaxed with judicious reserve, have evidently guided the proceedings of the Society which are about to become the subject of our consideration.

The present collection of Memoirs commences with the relation of an instance of—

*Successful Operation of Paracentesis of the Thorax;* by NICHOLAS ARCHER, M.D.

"In the month of October, 1798," says Dr. Archer, "I was sent for to visit a gentleman, aged 41, who, until within the last three years, had enjoyed very good health, was of a strong athletic form, and lived a temperate life.

"I could collect from his physician, that, about three years previous to my visit, he had an attack of pleurisy, which yielded to two general bleedings, a blister, and the antiphlogistic regimen. Shortly after his recovery from this attack (which seemed to be confined chiefly to the right side of the thorax), he began to complain of a short dry cough; a sense of heat in the right side, not amounting to pain; difficult breathing on using any exertion; palpitation; and lividity of countenance. When he remained quiet, his breathing was perfectly tranquil, and his countenance natural.

"He had consulted most of the medical men in London and in Edinburgh, whose several opinions coincided in recommending him to remove to a more temperate and southerly climate. According to the recommendation of these medical gentlemen, he went over to Lisbon, and thence into the interior of Portugal; where he remained more than a year, without reaping the smallest advantage by the change, which induced him to return home. Whilst in Portugal, he thought he could perceive the motion of a fluid in the right side of his thorax, and endeavoured to impress this idea upon his medical friends, whom he re-visited on his way home, but without effect.

"On my visit to him, which was nearly three years after his first attack of pleurisy, he complained of all the above-recited symptoms, but in a more aggravated degree; his countenance nearly approaching to dark lividity, and his pulse at 130 in a minute; great emaciation; but still his breathing was but little disturbed, except on exertion.

"He begged of me to examine him very particularly; for which purpose I placed him on his back, a little inclining to his right side, and pressed strongly with my fingers between the ribs of his right side. On his gently agitating his chest, I distinctly perceived an undulating motion under my fingers; and, on applying my ear close to the part, I could hear a noise like that produced by shaking a small cask not quite full of water.

"I told him, that his idea of the existence of water in his chest was well founded; that it would be most difficult to remove it by absorption, and recommended that it should be drawn off by operation. He submitted to the suggestion with the greatest alacrity; and, in a short space of time, two other medical gentlemen were procured, who agreed that nothing less than the paracentesis of the thorax could serve him.

"The operation was accordingly performed, and eleven pints of an inodorous fluid, resembling whey, were gradually abstracted. The tube of the canula was frequently obstructed by a solid substance, but, on the introduction of a probe, it used to pass off. On examining these solid substances, they were discovered to be small branches of the bronchiæ, in a shrivelled decayed state. During the drawing-off of the fluid, his pulse gradually diminished in quickness and increased in fullness; and, at the close of the operation, they rested at 86.

"For some few days after the operation, the discharge from the orifice amounted to nearly two pints in twenty-four hours, of the same kind of fluid; but it gradually lessened. His breathing became free; the lividity of his countenance disappeared; his appetite mended; he daily gained flesh; he was able, in a few weeks, to walk and ride out in the open air; and his cough nearly subsided.

"At his own suggestion, a slightly-astringent lotion was thrown into his chest through the open orifice; at first, consisting of a small portion of lime-water mixed with rennet-whey; and, in a short time, we ventured on a weak solution of sulphat of zinc in rose-water, with the most decided advantage. In four months all discharge nearly ceased; he gained strength, and enjoyed tolerably good health for three years.

"It would appear as if the first attack of pleurisy which this gentleman had suffered had been the cause of all his after symptoms. It is probable that an ulceration of the external pleura had taken place, and that a small lympathetic had been included in this ulceration, which, gradually oozing its lymph into the right cavity of the chest, occasioned a gradual compression of the lungs; which, in the course of the progress of the disease, must have been compressed into a very contracted space. It would appear, too, as if the lungs themselves were free from ulceration, as he had but little cough, and but very little expectoration, which was perfectly mucous, and not approaching to the nature of the fluid drawn off.

"Eleven pints of fluid would nearly occupy the whole of the right cavity of the chest; therefore, there could be no space at that side for the lungs to expand in inspiration. He had no œdema."

We have transcribed the whole of the observations of Dr. Archer on the above case, and have to regret that a studied conciseness of relation appears to have deprived us of some important information respecting it. It is very desirable to know whether or not the right lung became distended, and performed its natural functions, after the operation: this we think might have been ascertained from obvious means of experiment. The changes that took place in the pulse, breathing, and countenance, after the fluid was drawn off, seem to indicate that it admitted of the transmission of blood; whilst the length of time that it had suffered such powerful and extensive compression, and the separation from it of "small branches of the bronchiæ, in a shrivelled decayed state," would lead us to suppose that it had undergone a change of structure analogous to what is observed in other organs, on many occasions, when their functions have been long suspended, and they have become, in a manner, extraneous parts in the animal economy. Physiological reasoning would lead us to consider the consequences of the operation as essentially connected with the above circumstances. Without a knowledge of them we are, therefore, unable to determine either the precise inferences of practical utility that may be deduced from it, or the rank that should be assigned to this, amongst the few instances that are recorded in which a similar operation has been attended with fortunate results. We should rather attribute the serous effusion to chronic inflammation of the pleura, than the cause assigned for it by Dr. Archer.

There are, however, some circumstances of much interest attendant on the above case and the measures employed by Dr. Archer. They will assist in removing the undue fears that have been entertained respecting the exposure of the cavity of the chest to the external atmosphere; and, in conjunction with the celebrated operation of M. Richerand, and the opinions he has advanced respecting a new mode of treating many diseases of the thorax, particularly effusion of serum into its cavities, may probably furnish grounds for some useful practical improvement in the art of surgery. The operation performed by the people of the Tonga Islands, as described in the narration of Mr. Mariner, the authenticity of which can be relied on, should also not be neglected in our reflections on this interesting subject.*

II.—*Two Cases of fatal Constipation of the Bowels.* Communicated by William Stoker, M.D.

"These cases," Dr. Stoker observes, "are not uncommon; nor probably would the disorganization found after death appear so, if examination of the bodies of those dying of such diseases was more general." They will be contemplated with considerable interest by the more reflective part of the profession. Our limits will only permit us to observe, that in one case, that of a lady seventy years of age, where the patient had been long subject to obstinate costiveness, and at length died in consequence of this affection, the transverse arch of the colon was found much distended and ruptured; apparently in consequence of a diseased state of the rectum, which was hard and contracted for the space of four inches.

In the other patient, who died under analogous circumstances, the transverse arch of the colon, and the cœcum, were much distended: the latter would contain nearly a

* See *London Medical and Physical Journal* for February.

gallon of fluid, and a small aperture was discovered on a part of its surface. "The uterus was much enlarged and hard, and evidently cancerous. It had formed firm adhesion to the rectum and bladder, both of which were in some degree affected by the cancerous disease." The rectum was almost entirely obliterated for a space of nearly six inches.

III.—*Case of Ruptured Uterus;* by CHARLES FRIZELL, M.D.

Dr. Frizell has judiciously considered that every instance of favourable termination of so serious an accident merits publicity; for although, as he observes, cases of this kind are related by Dr. Stanton, Dr. Douglass, Mr. Manning, Dr. Hamilton, Dr. Joseph Clarke, Dr. Labat, and many other physicians, yet the fatal termination of the greater number of them would lead practitioners almost to despair of a favourable event, and in some degree prevent the active exertions that should be made for the assistance of the patient.

In this case the woman, the mother of six children, had been in labour during three days and two nights, when she was visited by Dr. Frizell: she was then much exhausted. An arm of the child presented in the vagina: the left foot was brought down, but the attempts to deliver the fœtus by it were not successful. Hæmorrhage now came on. On further attempts, the leg separated; and, on introducing the hand in order to bring down the other, a portion of the intestines was discovered in the vagina. With the help of a crotchet affixed to it, the right foot was brought down after some difficulty, and the child wholly delivered. A rupture of the uterus was found "to commence at the upper part of the symphysis pubis, and to proceed, on the left side, to nearly the middle of the opposite part of the sacrum."

"I carefully pushed up the intestines," continues the author, "but found some difficulty in replacing them, as they constantly protruded, or one portion presented itself whilst I was endeavouring to replace another. Under these circumstances, I tried to bring the lacerated edges of the rupture together; but, still finding the intestines to protrude a little, I pushed them up pretty high, and, slowly withdrawing my hand, brought the edges of the rupture to overlap one another, and think, after this arrangement, they retained their situation, particularly as the womb had by this time contracted a little; and, on subsequent examination, I did not perceive that they had descended."

By a judicious application of mild antiphlogistic measures, the unfavourable symptoms were obviated: the patient finally recovered, and has since produced another child.

IV.—*A Case of an unusual Termination of Psoas Abscess.* Related by SAMUEL WILMOT, Member of the College of Surgeons in Ireland; one of the Surgeons of Dr. Stevens' Hospital, and of the Charitable Infirmary in Jervis-street; Lecturer on Surgery, &c. &c.

The patient of this case was a man, aged 22, who became affected with pain in his loins after having slept in a damp bed. A year afterwards, when he consulted Mr. Wilmot, he had a tumor in the groin about the size of a tennis-ball, evidently containing a fluid. There were also present hectic fever, and symptoms of psoas abscess, unaccompanied with disease of the vertebræ. He then refused to submit to the measures proposed by Mr. Wilmot. After another year had elapsed, he again requested his advice; when the tumor in the groin had much increased in size, measuring nearly fourteen inches in circumference at its base, and apparently containing a gaseous fluid, which could be made to pass into the abdomen by pressure. The canal of communication was beneath the femoral ring. This was the only inconvenience he suffered: his health was perfectly restored.

Mr. Wilmot adduces some reflections relative to this phenomenon, which we shall have occasion to illustrate on a future occasion, when we take into consideration the *Croonian Lecture* given by Sir EVERARD HOME to the Royal Society in the year 1818.

"We know," says Mr. Wilmot, "from numerous facts, that the cysts of abscesses are highly organized, having arteries whose office it is to secrete pus, and absorbents which not unfrequently take up, either totally or in part, the matter in contact with them. In the case under consideration, the matter was entirely absorbed; and there was also a cessation to the secretion of more matter, in consequence of the great change in the patient's constitution. The arteries, having ceased to be employed in the secretion of pus, now assumed the office of secreting air; and by this means the gradual obliteration of the cyst by contraction was prevented, which is usual in ordinary cases, when the matter is removed by the absorbents. There is no point in pathology better established, nor one more universally admitted, than the power of arteries to secrete or separate air from the blood. This is proved in cases of emphysema, where the lungs are entire, and also in that affection termed tympanitis: if, then, it is admitted that the arteries of the cellular membrane, and of the stomach and of the intestines, can at times secrete air, I conceive that I have not advanced any thing strange or inconsistent by allowing a similar power to the arteries of the cyst of an abscess whose integrity had not been destroyed, either by a natural or artificial process."

There were but two obvious modes of curing this affection, Mr. Wilmot remarks,

"One by puncturing the tumor and letting out the contained air, and then bringing the sides of the sac into contact, so as to cause the opposite surfaces to adhere, and in this way destroy the cavity; or by pressure, to promote its absorption, and, by a continuation of the pressure, to bring about obliteration, either by contraction or adhesion. I determined upon trying the effect of pressure, which succeeded most effectually. I applied a bandage and compress, wet with a strong decoction of oak-bark and alum: this application produced a considerable corrugation of the skin. In about three weeks the air was almost entirely absorbed, and the sac considerably diminished. I now got a truss, made with a pad so as to cover the entire extent of the sac. The patient was now able to walk; and, in about four weeks from putting on the truss, left the hospital perfectly free from any swelling."

V.—*On Apoplexia Cephalitica;* by Wm. Stoker, M.D.

Some facts that have occurred to the observation of Dr. Stoker led him to consider that advantage would arise from a subdivision of Cullen's second species of apoplexy into two distinct varieties,—the first to be termed *apoplexia cephalitica*, the second *apoplexia hydrocephalica;* and some cases are related in this paper, which, he considers, show the propriety of this arrangement.

The errors and incongruities of Cullen's nosological classification cannot fail to engage the attention of every observant and judicious physician; especially those parts of it where the remote consequences are confounded with, or considered as, the proximate effects of disease; which parts have been productive of more injury in their application in practice, by those who attend to words rather than the real ideas of things, than could possibly have arisen from the total want of any classification of morbid affections.

A *methodical* arrangement of diseases is certainly a very desirable object, and is, indeed, essentially necessary to the perfection of the practice of medicine; but it is not on such a foundation as the system of Cullen that this can possibly be raised. Our ideas of the nature of diseases must, it is evident, be derived from their symptoms and signs; for their proximate causes, like those of physical phenomena in general, will never be discovered: we can only trace proximate effects, and it is from an accurate observance of these, their origin, progress, mutual and relative analogy or incongruity, and *their* effects, that all our real knowledge of maladies must be derived. This is a task of the utmost difficulty, and one that requires an acute and philosophical mode of enquiry ; but much progress has already been made in it, and that through the most difficult part of the necessary course : the period, we expect, too, is not very distant, when it will be so far completed as to render the healing art an absolutely beneficial application of the powers of the human intellect. Cullen's system, it is true, is founded on the symptoms of diseases, but, secondary, and even more remote, consequences, are often argued on as proximate ones ; and thence the serious errors it leads to in its application in practice. The most eminent modern pathologists consider morbid actions as modifications of healthy ones ; that is, that diseased actions are merely variations of essentially existing natural functions : if this be true, no real knowledge can be acquired respecting any one disease in particular, nor any correct methodical nosology be framed, except on physiological principles : a consideration totally neglected in the greater part of the system of Cullen.

As long, however, as that system is generally adopted in our schools, every attempt to improve it must be contemplated with pleasure.

The cases related by Dr. Stoker show that the ordinary symptoms of apoplexy will arise from increased afflux of blood to the brain, or inflammatory excitement of that organ, and exist without the extravasation of either blood or serum. This is a fact that has been frequently observed, but it cannot be too often presented to the attention of medical practitioners whilst erroneous ideas respecting the nature of such affections continue to be associated with the names by which they are designated by systematical writers. The proposal of Dr. Stoker is not free from objections ; for, in the first place, it would perhaps be better to confine the term *apoplexy* to sudden deprivation of sense and voluntary power ; and it is evident that what is apoplexia cephalitica one day may be apoplexia hydrocephalica the next ; and that serous effusion and inflammatory action may exist at the same time. But we must quit this subject, notwithstanding its importance ; for we perceive that we are only repeating the remarks of Dr. Kinglake that were inserted in the last Number of this Journal.

VI.—*An Essay on Dreaming ; including Conjectures on the proximate Cause of Sleep* ; by ANDREW CARMICHAEL, M.R.I.A.

This is an essay which we can only point out to our readers as worthy of their attentive consideration, as it will not admit of an analysis adapted to the limits of our Journal. It contains numerous facts relative to psychology, metaphysics, and physiology, of the most interesting nature to all who have a disposition to philosophical contemplation. The reader will, however, encounter some propositions in it that will, perhaps, cause surprise on a first, or superficial, view, but of which a little reflection will demonstrate the apparent truth ; he will also meet with many notions that will not, probably, be admitted to be correct until some years shall have elapsed. There are some points in it that appear to admit of dispute, but we cannot notice them here, as it would not be right to do so without adducing the arguments of the author in their support.

It is a subject for regret that this essay has not yet been published in a separate form : it should not escape the attention of any psychologist, or metaphysician, and there are many who would not expect to find so fine a specimen of the *καλοκαγαθον*, on such a subject, amongst a collection of Medical Memoirs.

*(To be continued.)*

*History and Description of an Epidemic Fever, commonly called Spotted Fever, which prevailed at Gardiner, Maine, (in the United States,) in the Spring of* 1814; by E. Hale, jun. M.D. M.M.S.S.—Wells and Lilly, Boston; and Souter, London. 1818. 8vo. pp. 246.

Those who have witnessed the progress of a desolating epidemic disease, especially one of a novel character, or of which the origin is unknown, can alone correctly appreciate the value of faithful histories of that class of maladies, and feel of what importance it is that none of them should be suffered to pass by unnoticed in the annals of medicine. Without such histories, the physician knows that he must see many persons fall victims to their influence, who might have been saved had he possessed the results of the experience of others under similar circumstances; and, although the judicious practitioner may soon discover the means best adapted for their relief, yet he cannot with equal precision ascertain the essential cause of their production, and thence determine efficient prophylactic measures, during the consternation attendant on their ravages. This knowledge, in the greater proportion of cases, can only be acquired from the consideration of their phenomena, as evinced in different countries, climates, seasons, and under various modes of social intercourse: and even with this information, we have been enabled to determine it only in a very few instances. The essential cause of the greater part of them is not better known at the present time than at the earliest period of which we have any historical record: we still assign to most epidemic diseases the same origin that Homer did to that which afflicted the Grecian army, encamped on the sea-coast, at the siege of Troy,—the influence of the rays of an ardent sun on a marshy soil.—

τοῦ δ' ἔκλυε Φοῖβος Ἀπόλλων·
Βῆ δὲ κατ' οὐλύμποιο καρήνων χωόμενος κῆρ,
. . . . . . .
Ἕζετ' ἔπειτ' ἀπάνευθε νεῶν, μετὰ δ' ἰὸν ἕηκε.

It appears probable that some ages will elapse before the primary cause of the epidemic maladies with which we are already acquainted, can be decidedly ascertained. To illustrate the truth of this remark, we need only to refer to the yellow fever. Although we possess histories of that disease, by men of considerable talents, from the year 1741 (when it appeared in Virginia) up to the present period, during which interval it committed continual ravages throughout the greater part of the globe, still there exist different opinions respecting its origin amongst men of extensive experience and of the highest repute for the possession of professional knowledge.

Whilst then, as Homer expresses it, the effective agents of epidemic diseases take their course in the shades of night, we must collect with assiduity whatever appears to be connected with their development: we may thus supply to future generations the means for acquiring that knowledge which we ourselves have not been able to obtain.

The subject of the work of Dr. Hale will be contemplated with sensations of particular interest in this country; for, although that disease may have occurred only in a remote quarter of the world, and appears to have totally ceased to exist, yet, the similarity of the climate, in many parts where it appeared, to that of our island; the identity of the race of the greater proportion of the subjects of it with that of the English people; and some analogy that appears to exist between it and maladies which at different times have ravaged our own country, (as far as the imperfect accounts we have of them will enable us to judge,) give rise to reflections that show the forcible claims it has on our attention.

Our object, then, in taking up the work of Dr. Hale, is to point out what will complete the history of the spotted fever of North America up to the latest epoch of its appearance, and to show its character under different circumstances of topical situation and habits and conditions of those who were the subjects of its influence. We have already fully described it as it existed in the years 1810, 1811, 1812, and

1813;* and have, therefore, only to point out such useful additions to our knowledge respecting it as are supplied to us by Dr. Hale.

The author, in a Preface, observes that—

"Several treatises upon the spotted fever have already been published in this country: but, as their object has been to give such an account of it as would apply to its general character as it appeared in different places, they could not, of course, take notice of many of the modifications which it acquired from various local circumstances. It has been my object, in this volume, to give a more *clinical* view of the disease; to exhibit it in its varieties, as it appeared to the physician at the bedside of his patient; rather than to seek its place in a regular system."

That this has been well effected by the author is evident from the marks of acuteness and precision of observation, and comprehensive and judicious views, that are manifest throughout the work; which must, from these circumstances alone, prove highly valuable to the practitioner, should the subject of it recur at any future period.

Dr. Hale commences with a topographical description of the scene where the spotted fever occurred to his observation; he next describes the habits and manners of the inhabitants, the diseases generally prevalent amongst them, especially those with which they were affected for a short time before the existence of the epidemic, of the origin and progress of which he then proceeds to give a particular and general history.

The face of the country throughout the district of Maine is for the most part hilly, though rarely mountainous; the valleys between which extend only a short distance, soon rising to the elevation of the surrounding country, which is much higher than the elevation of the rivers, with which the whole of this district is well supplied. The parts of it extending to the sea-coast are generally rocky, and apparently barren. The interior is for the most part abundantly fruitful; the soil of which is in some few places sandy, more frequently clayey, and still more extensively, loamy. The towns have been all recently settled, very few of them being more than forty years old, and most of them still more modern: of course, extensive forests prevail in every part of the district. The climate varies in temperature from a range of the thermometer, of from several degrees below the zero of Fahrenheit, to 80 or 90 above it; but it is much less subject to frequent and violent changes of temperature than

* See *London Medical and Physical Journal*, vol. xxvi. p. 217; vol. xxix. p. 328; and vol. xxxiii. p. 92.

the more southern parts of the country. The winter is long, and the transition from that to summer is rather sudden. Rains, which are rare in winter, are generally sufficiently abundant in summer. Violent winds are exceedingly uncommon, and in cold weather never occur. "Nothing can exceed the serenity, transparency, and brilliancy, of a cold winter's evening on the Kennebeck." The town of Gardiner is situated on the west side of the river Kennebeck, about forty miles from its mouth, in north latitude 44° 14′, and west longitude 69° 44′. The inhabitants are generally farmers; and many of them, having been long accustomed to obtain their support from the produce of the forest, are hardly reclaimed from the irregular and improvident, though hardy, habits, to which their mode of life had formerly subjected them.

"From the preceding observations," observes Dr. Hale, "it will naturally be inferred that the diseases to which they are most subject are those of an inflammatory kind. This may be true in general, although, during the time I resided in Gardiner, it was only to a very limited extent in that place and its vicinity. Rheumatisms, especially chronic rheumatisms, were very common; but, excepting these, diseases of inflammation were exceedingly rare, and in those which occurred there was such a tendency to prostration of strength, that much caution was necessary in the use of depleting remedies. Almost all cases of fever, which I saw, partook more or less of the character of that described in this treatise."

After some judicious reflections on the importance of attending to the state of the atmosphere, &c. as connected with prevalent diseases, which our limits will not permit us to notice; and a detail of the cases that occurred in his practice immediately previous to the appearance of the epidemic, which we pass over as not furnishing any apparent data at all connected with it; the author enters into the history of the spotted fever.

"At the commencement of the year 1814, there was nothing at Gardiner to indicate the approach of the epidemic that was to follow, unless it was its prevalence in some towns in the vicinity. The year preceding had been abundantly fruitful. The autumn and first part of the winter was drier than usual, but not so much so as to produce a drought of any importance. The winter was a pleasant one, without any unusual physical occurrence to distinguish it from others in that climate.

"Early in the autumn of 1813, we began to receive accounts of a destructive epidemic in many towns not far distant. As the winter advanced, the accounts became more and more threatening as the disease approached nearer to us. It was frequently fatal, and the character which it acquired by report did not diminish its terrors. The first case in Gardiner, to which I was called, was on the 11th of February. The patient had been several days ill, but not so sick as to call in a physician till this time. The case proved to be a severe one, but eventually terminated in recovery. It was nearly a fortnight before any other cases of the fever occurred. Towards the last of February, however, several attacks followed each other in such quick succession as to produce a considerable alarm: some of these were in the family and immediate neighbourhood of the person first seized; others were at a distance, and had had no communication whatever with the sick.

"Throughout the month of March the epidemic extended itself rapidly in all directions. In some of the families, where it first made its appearance, almost every person was seized by it; in others, only one or two were at any time materially affected: in some cases it seemed to spread progressively from house to house, as if communicated from one person to another; at the same time that in others it suddenly made its appearance in distant neighbourhoods, seizing sometimes two or three persons in a family, nearly at once. All classes of people and all ages seemed alike exposed to its attack.

"Towards the end of this month the epidemic was more prevalent than at any other period: within a small circuit, more than fifty were confined with it at the same time; many others, who were not reckoned among the sick, were slightly affected by similar complaints; so that the sick and the invalids included a very large proportion of the population.

"Early in the month of April the progress of the epidemic began to abate, and it continued to diminish throughout that month, especially in the parts of the town in which it had previously raged. About the 20th, I was called to a considerable number of cases in Pittston, on the east side of the Kennebeck river; as well as to several new cases in Gardiner.

"Throughout the month of May, also, a considerable number of cases occurred; but they grew less and less frequent until the close of the month. The epidemic may be said to have terminated its course in Gardiner within this month. In each of the three following months, of June, July, and August, I did not see more than two or three cases of fever of any kind.

"During the whole period of the epidemic, sores of different kinds were unusually prevalent, as well as for some time after its termination. The most frequent of these was a species of boil, somewhat resembling a carbuncle, which was very common with the convalescent, as well as with those who had not been affected with general fever. It was a very painful tumor, which, in the course of two or three days from its commencement, ulcerated, and cast off a gangrenous slough. They were not often so severe as to require any other medical treatment than an emollient poultice, except when they were merely symptoms of a more important disease. The whitlow was also unusually prevalent at this time. Headachs and other slight symptoms of fever were almost universal. Hardly a person could be found in the village of Gardiner, or its immediate vicinity, who had not, in the course of the three sickly months, been the subject of an affection more or less severe, which was similar in its character to the more important cases of fever. Most of these, perhaps, would hardly have been noticed at any other time; but they deserve to be mentioned as examples of the strong and universal tendency to a particular disease, which prevailed at that period.

"It was observable that the epidemic, throughout its whole course, was remarkably affected by the state of the weather, and especially by any sudden change in its temperature. This was true, not only in respect to the effect on individual cases, but also as applicable to the epidemic as such. A few days of unusual cold seemed to render all the existing cases more severe, and at the same time produced a greater number of new attacks; while, on the contrary, a change from cold to milder weather produced a corresponding effect, in mitigating the symptoms and lessening the ravages of the disease."

We shall pass over the description of the symptoms and progress of the disease, as these did not materially differ from them as related in former parts of our Journal, and our limits will not permit us to copy at length the numerous traits of acute discrimination, on which the peculiar excellence of the present work depends.

It is much to be regretted that Dr. Hale had not opportunities to make examinations of its subjects after death. As far as they were carried on former occasions,* it would appear that cerebral, and sometimes pulmonary, congestion, occurred to an extraordinary degree, in the first instance; and that the patients frequently fell victims to the immediate consequences of compression of the brain, before disorganization of any part from inflammation had commenced. In those who survived the attack a few days, the consequences either of inflammation of the brain or of the serous membranes of the pectoral and abdominal cavities, were constantly observed. The state of the mucous membranes is not noticed; and probably they were not accurately examined, for it is but rarely that they are sufficiently attended to in *post mortem* dissections. The greater proportion of those who fell victims to it died before the inflammation could, according to the general operations of the animal economy, extend to those parts; and, consequently, before febrile re-action took place. We, nevertheless, consider extreme excitement of the brain as the immediate cause of the disease, although this does not concur with the ideas of the author; and our opi-

* See *London Medical and Physical Journal,* loc. cit.

nion on this point is not shaken by the results of the remedial measures that were considered by Dr. Hale to have been most efficacious,—which were chiefly emetics, the pediluvium, diaphoretics, and active stimulants. These, it is obvious, would act as counter-irritants, and might be productive of benefit, when employed before general febrile re-action commenced; although we consider the use of them as somewhat hazardous.

"The first and leading object (observes the author,) always was to restore, and continue in force, the functions of the skin. The second, which was hardly less important, was to support the strength of the patient. The remainder of the cure was effected by removing the great variety of occasional symptoms which occurred. The means for accomplishing the two first objects were pretty uniformly the same in the several cases; but, for the last, the whole materia medica presented a field hardly enough variegated for the complicated and perpetually changing evils to be removed.

"At the beginning of the epidemic season, I pretty generally commenced the treatment by administering an emetic; but not finding, in most cases, the benefit from its operation which I had anticipated, I soon omitted it, except in cases where there had been symptoms of a derangement of the functions of the stomach previously to the attack of fever. In these cases, an emetic at the commencement of the disease was of very great service, and sometimes entirely arrested its progress.

"Before the emetic was given, however, the patient was put into bed, and pretty commonly had made use of the warm pediluvium."

Powerful diaphoretics were then given; and he continues to observe—

"If the limbs were cold or numb, or subject to pain, directions were given that they should be diligently rubbed, either with the naked hand or with flannel, either dry or moistened with oil or with some stimulating liquid,—such as vinegar or alcohol, and sometimes with a solution of cantharides.

"In this manner the cure was always begun; and, in cases in which the strength was not particularly depressed, very little else was prescribed at the first visit, except an anodyne at bed-time. In the first part of the season particularly, when the pulse was often considerably full and strong, and especially if there were symptoms of a pneumonic affection, I waited until these symptoms had somewhat remitted before I began to administer the tonic remedies, which held a conspicuous place in the general plan of treatment. But when, as in a great proportion of cases, the strength was low from the first, or if it had become so by the continuance of the disease, it was necessary, in addition to the treatment already described, to take vigorous measures to prevent it from sinking altogether: for this purpose small quantities of brandy were occasionally given in the drinks already mentioned, a diet as nutritive as the patient could take was recommended, and a variety of medicinal tonics prescribed.

"When symptoms of faintness or torpor appeared, at whatever period of the disease it might be, the diffusible stimuli were diligently administered. The aromatic spirits and volatile oils, in all their variety, were given in small doses frequently repeated."

These measures were successful in by far the greater proportion of instances; and, therefore, it would be vain to adduce theoretical opinions against the remarks contained in the following paragraph:—

"I mention venesection (says Dr. Hale,) among the remedies for this disease, although I did not employ it myself, nor see any case in which it had been employed; because it has generally been considered a powerful remedy, and because it gives me an opportunity to say that I have had no experience of its efficacy. I was deterred from practising it by the great tendency to debility which I witnessed in the disease, as well as by the reports which I had heard of the disastrous effects which were said to have followed its use in other places. The foundation of these reports, or the accuracy with which they were related, it does not come within my plan to examine here."

Should, however, a similar disease appear in our milder climate, and amongst its more plethoric inhabitants, we should advise the pediluvium to restore some degree of re-action in the extremities, if torpor to the extent that occurred in the epidemic under consideration should take place; and then that blood-letting should be used with freedom, without being alarmed by the debility, langour, stupor, coma, or "exhaustion of the vital powers:" for we consider these symptoms to have arisen from compression of the brain, and the abstraction of the natural excitement of the body in general, consequent on the great irritation of that organ. Under some circumstances, such measures, however, as will cause diffusion of an equable excitement may, doubtless, be the most prompt and efficient means of relief; and such would appear to have been the case in those witnessed by Dr. Hale.

Our opinions on this subject are supported by those of the committee formed at Massachusetts, in the year 1810, when a similar disease was epidemic in various parts of New England; as the measures we have pointed out coincide with those inculcated by the enlightened members of that committee.

We must refer our readers to the author himself for his observations on the remedial treatment which he considered best appropriate to this disease, and for the cases which he relates to illustrate the efficacy of that which he employed.

The work concludes with some general remarks on the nature of the disease, and the peculiar character it has assumed under different circumstances of temperature of climate, topographical situation, and the habits of those who were the subjects of its influence. But the same reasons we gave for not entering into the particular consideration of its symptoms, as it occurred to the observation of Dr. Hale, will equally apply to the points to which we have just alluded. We cannot, however, with propriety, dismiss this work without remarking that, it will constitute a valuable clinical guide to the medical practitioner who may be called to witness the recurrence of the epidemic; and it merits a place in every medical library, as a perspicuous and accurate history of a malady, which, under the name of the *spotted fever*, has inspired a dread throughout the United States that will scarcely be forgotten so long as memory or tradition shall continue to exist.

*An Essay on Catarrhal Inflammation of the Intestines from Cold;* by JOSEPH ASHLEY GAITSKELL, M. D. Junior Physician to the Female Penitentiary and Lock Hospital, Bath.—1819.

THIS little essay treats with perspicuity on a disease of frequent occurrence, but not sufficiently discriminated in general systems of practical medicine. It is confounded, under the common name of diarrhœa, with affections of different origin and character. The author, following in a great measure the suggestion of Dr. Parr, proposes to rank it under a genus which should be designated by the term "Catarrhus," to be applied to inflammation of mucous membrane attended by symptomatic fever. The species of this genus would be marked by the individual membrane which became the seat of disorder. Under this system, the disease which forms the subject of Dr. Gaitskell's immediate consideration is denominated *catarrhus intestinorum*. This classification appears to us conformable with the legitimate end of nosology, the distribution of identical facts of pathology under distinctive and appropriate heads. Similar affections of resembling structures form the natural basis of nosological arrangement, and, if more strictly attended to, would have afforded systems of more permanent form than have heretofore been framed, and better fitted to impart precision to our notions of disease.

The disease under present notice is characterised by—

"Pain of the belly in the region of the bowels, often extending into the back, aggravated by pressure or change of position, and sometimes by deep inspiration; much eased by quietude and recumbent position. Belly soft. Stools frequent, watery, and acrid; so much so, as in many cases to excoriate the anus. When the small intestines are much affected, sickness and head-ach are prominent attendants; when the large intestines, tenesmus. Fever 'of the catarrhal kind.' From the function of the part, the secondary condition of the secretion is seldom observable in practice."

The author goes through the history, causes, diagnosis, prognosis, and treatment of the disease, with all requisite attention to the method of systematic writers, but perhaps somewhat more diffusely than the general importance of the subject would call for. In his diagnosis are some judicious discriminations, thrown into a tabular form, betwixt this particular species of inflammation of the intestines and other diseases of the same parts, more serious in their character.

*A Treatise on Midwifery; developing new Principles, which tend materially to lessen the Sufferings of the Patient, and shorten the Duration of Labour;* by JOHN POWER, Accoucheur, &c. Member of the Royal Medical Society of Edinburgh. pp. 270.—London, T. and G. Underwood. 1819.

THE title of this book will, we are sure, fix the attention of many of the profession; and, amongst the readers which it will doubtless procure for the work, we will venture to say that few will be dissatisfied with the time devoted to its perusal. We do not presume to say that the expectations raised by the title-page will be amply fulfilled; experience alone can give warranty to the assurances there advanced; but we are convinced that the order, clear induction, and enlightened views, which pervade this volume, will ensure for it a considerable share of public approbation; and, for the opinions of its author, the fair meed of general respect.

Mr. Power does not profess to embrace the full consideration of all that is usually comprised in systematic treatises on midwifery; on the beaten path he touches cursorily, and only dwells on those parts where he would point out some novelty of view or improvement of arrangement. With this view he treats briefly on the anatomical structure of the parts concerned in parturition; on the physiology of the parturient process he dilates somewhat more at length. Considering pain as an adventitious concomitant of uterine action, incident to the human female, probably, from the habits of artificial life, he would exchange the designation of the expulsive effort of the uterus, labour-pain, for parturient paroxysm. This, in its perfectly natural state, is stated to be simply muscular action of the organ itself, not interesting other parts, and depending, as does the action of other muscles, on influence imparted from the nervous system. This peculiar modification of nervous power is termed the parturient energy. We merely give these definitions of terms and general positions, as explicative of the author's language in the further progress of the work.

On the exciting cause of uterine contraction, at the term of utero-gestation, the reasoning is very satisfactory. Mr. Power refers it, on analogy from the action of other expellent organs, to an impression made on the orifice of the viscus by the contained matters, which, at the period when the uterine nurture of the fœtus is complete, are, by the expansion of the cervix, brought into immediate contact with it. Reasoning from the derivation and extent of the nervous structure of this part of the organ, he says—

"It may hence be inferred, that the orifice of the uterus possesses a high state of nervous power, and consequently a peculiar function. It has also been observed, that this part becomes little connected with utero-gestation until that office is complete, being previously removed to a determinate distance from the distending process. Is it not, therefore, reasonable to consider that its peculiar function, so far as is connected with a high state of sensibility, is to give warning of the task of utero-gestation being perfected; and to be the medium of calling into action the powers which are appointed to produce the expulsion of the now mature fœtus?"

The establishment of this point is of some practical importance; as, from the knowledge of it, we are enabled, by applying artificial irritation to the part in question, to command in some measure the expulsive action of the uterus.

On entering upon the pathology of parturition, the deviations which occur from a perfectly natural condition of the process are distributed into—

"*a.* Deviations arising from the state of the parturient energy.

"*b.* Deviations produced by mechanical obstruction to the expulsion of the uterine contents.

"*c.* Deviations arising from accidental circumstances."

The section which treats of the first of these orders of irregular parturient action is, perhaps, one of the most important in the book, as in it are developed, in a great measure, the more peculiar views of the author.

He classes the derangements of the parturient energy under the following heads:—

"*a*. The parturient energy, although it evinces perfect uterine action, produces spasmodic pain in the organs of parturition.

"*b*. The parturient energy excites partial or irregular contractions of the uterine muscles.

"*c*. The parturient energy, instead of actuating the uterine muscles, excites actions of parts distinct from the uterus.

"*d*. The parturient energy is suspended, so that it ceases to actuate any part of the uterine or general system."

Under the first head of derangements, Mr. Power proceeds to show that the muscular structure of the uterus is like other muscles of the body, obeying the same principles and regulated by the same laws; and that, therefore, the ascertained facts of one set of organs may be applied in illustration of the functions of the other. In the natural state of contraction of a muscle, the exertion, though violent, is unaccompanied by pain; but, where long-continued opposition is made to the action of a muscle, or it becomes morbidly affected, spasmodic action of its fibres may be induced, attended by painful sensation, which is a symptom merely of the spasm. These principles, applied to the muscular action of the uterus, lead to the inference that the healthy contractions of that organ are, like those of other muscles, unattended by pain. This seems to be the case generally in the parturient efforts of the inferior animals; and that it is sometimes so with the human female is known, in cases of the uterine action going on during the sleep of the individual. From this it would seem that a state of spasmodic action in the uterus is as unnatural as in any other muscle. This preternatural state of action is referred to two causes: over-action, excited by the necessity of overcoming great resistance; and a morbid state of the constitution of the organ. Both these circumstances are considered at some length, and their influence demonstrated.

The second order of derangements, as of rare occurrence, is less important; but that partial and irregular action of the uterine fibres does sometimes take place is evinced in the hour-glass contraction, and may sometimes be discovered during the earlier stages of parturition, by the hand applied on the abdomen, when the uterus may be felt partially tense and in part flaccid. This state depends on some morbid or deficient determination of the parturient energy, or on some peculiar modification of the uterine muscles themselves.

The third order of derangements comprises considerations of much interest; as under this head we find the most frequently occurring causes, independent of malformation or disproportion, of protraction and of suffering in labour: and to this part of the author's work we are disposed to yield our warmest praise. It is a high satisfaction to follow a lucid development of principles rendered almost demonstrable; and it is a grateful task to acknowledge the conviction which such a train of reasoning imparts.

Mr. Power premises some general views of the nervous energy, which regard its homogeneity, diffusibility, and instantaneity of local determination, affording strong analogies with the electric fluid, to which, he observes, it appears nearly allied. The quantity of its production is under limitation, and thus, if superabundantly determined to any given part, it must become comparatively deficient in other parts of the system. By this law of the distribution of the nervous power, many of its phenomena are to be explained.

"Amongst other effects, it is a consequence of the above law that a secondary action, excited in the system, is capable of counteracting the pre-existent one, so as to diminish, and even entirely supersede, its operation.

"It is here conceived, that the nervous energy supplying the primary part is superseded in that part, in consequence of its being determined, by virtue of a superior irritation, to the secondary one, which then becomes actuated.

"The principle has been termed metastasis, because a translation is made of the actuating nervous power from the primary to the secondary part."

Metastasis is sometimes the effect of a counter irritation affecting the secondary part, whereby the excitement in the primary part is lessened or superseded. This may be considered a state of direct metastasis; but there is another state of metastasis dependent on the consent of parts through nervous influence, implied by the term sympathy. Thus an irritation may be applied to a given part, and a consequent action is not only induced in a distant or secondary part, but the primary one ceases to be affected, although the irritating cause may continue to be applied to it. This state may be distinguished as a sympathetic metastasis, being independent of direct irritation applied to the secondary part, but being excited through sympathetic communication. The sympathetic associations of the uterus are acknowledged.

"We also assert, that, during parturition, it is peculiarly susceptible of the metastatic state, of both the direct and sympathetic kind; and, had we nothing but the above analogies in support of this assertion, it is conceived that it might fairly be presumed. The opinion does not, however, rest on such imperfect ground, but may be fully demonstrated by actual fact and observation."

Assuming the tense state of the uterus, discoverable on applying the hand upon the abdomen of the patient during a parturient paroxysm, as the true criterion of efficient uterine action, it may often be perceived that, even during an acutely painful paroxysm, this tense feel of the uterine tumor is altogether absent, and the parietes of this viscus remain flaccid and yielding to impression. The os uteri, too, on examination, will be found entirely unacted upon.

"As it is clear that the action of such paroxysm, the effects of which are most decided and distressing, and which it cannot be doubted is produced by the influence of the parturient energy, is not expended upon the uterine muscles, so as to give rise to any real parturient effort, it must follow that the parturient energy which has produced it has, in consonance with the laws of metastatic action, been determined from the uterus, which it ought to have actuated to a distant or different part, the latter having been thrown into action, while the former has become quiescent.

"The above inefficient state is, therefore, to be considered as consisting of a translation of the parturient energy, from the uterine system, to a distant part.

"This translation may be effected either by direct or by sympathetic excitement."

This is elucidated by instances of metastatic action, occurring from irritation of the urinary bladder, determining the nervous energy to this viscus, or transferring it by sympathy to more remote parts.

"In all instances the parturient energy is equally diverted from the uterine muscles, and eventually determined by metastasis to the part ultimately affected, and thrown into inordinate action. As no practical advantage will be derived from further distinction, their future consideration will be conducted under the general term of metastatic action.

"Under a full state of metastatic action, the progress of the case is arrested, nor can it make the least advancement during its continuance, did this last (were it possible) *ad infinitum.* The author has known repeated instances where the delivery has, in consequence, been retarded for two, three, or more days, without the least progress throughout this long period of delay; notwithstanding, from the rapid advancement in the early stage, an expectation had been formed of a speedy termination: in some of these instances, when at length the genuine uterine action was restored, the child has been expelled in a few minutes, without interference on the part of the accoucheur. The same difficulties and similar terminations must have occurred to every experienced practitioner.

*(To be continued.)*

